# IL-2 / α-IL-2 Complex Treatment Cannot Be Substituted for the Adoptive Transfer of Regulatory T cells to Promote Bone Marrow Engraftment

**DOI:** 10.1371/journal.pone.0146245

**Published:** 2016-01-05

**Authors:** Benedikt Mahr, Lukas Unger, Karin Hock, Nina Pilat, Ulrike Baranyi, Christoph Schwarz, Svenja Maschke, Andreas Michael Farkas, Thomas Wekerle

**Affiliations:** Section of Transplantation Immunology, Department of Surgery, Medical University of Vienna, Währinger Gürtel 18–20, 1090, Vienna, Austria; Université Paris Descartes, FRANCE

## Abstract

Cell therapy with recipient Tregs achieves engraftment of allogeneic bone marrow (BM) without the need for cytoreductive conditioning (i.e., without irradiation or cytotoxic drugs). Thereby mixed chimerism and transplantation tolerance are established in recipients conditioned solely with costimulation blockade and rapamycin. However, clinical translation would be substantially facilitated if Treg-stimulating pharmaceutical agents could be used instead of individualized cell therapy. Recently, it was shown that interleukin-2 (IL-2) complexed with a monoclonal antibody (mAb) (clone JES6-1A12) against IL-2 (IL-2 complexes) potently expands and activates Tregs *in vivo*. Therefore, we investigated whether IL-2 complexes can replace Treg therapy in a costimulation blockade-based and irradiation-free BM transplantation (BMT) model. Unexpectedly, the administration of IL-2 complexes at the time of BMT (instead of Tregs) failed to induce BM engraftment in non-irradiated recipients (0/6 with IL-2 complexes vs. 3/4 with Tregs, p<0.05). Adding IL-2 complexes to an otherwise effective regimen involving recipient irradiation (1Gy) but no Treg transfer indeed actively triggered donor BM rejection at higher doses (0/8 with IL-2 complexes vs. 9/11 without, p<0.01) and had no detectable effect at two lower doses (3/5 vs. 9/11, p>0.05). CD8 T cells and NK cells of IL-2 complex-treated naïve mice showed an enhanced proliferative response towards donor antigens *in vitro* despite the marked expansion of Tregs. However, IL-2 complexes also expanded conventional CD4 T cells, CD8 T cells, NK cells, NKT cells and notably even B cells, albeit to a lesser extent. Notably, IL-2 complex expanded Tregs featured less potent suppressive functions than *in* vitro activated Tregs in terms of T cell suppression *in vitro* and BM engraftment *in vivo*. In conclusion, these data suggest that IL-2 complexes are less effective than recipient Tregs in promoting BM engraftment and in contrast actually trigger BM rejection, as their effect is not sufficiently restricted to Tregs but rather extends to several other lymphocyte populations.

## Introduction

Interleukin-2 (IL-2), originally discovered in the supernatant of activated T cells [1], was first described to drive the clonal expansion and effector development of antigen activated T cells [2,3]. Later it was recognized that IL-2 has also a critical function in down-modulating immune-responses as mice with a defective IL-2 pathway developed lymphoproliferative and autoimmune disorders [4,5]. This unexpected finding was soon ascribed to the critical function of IL-2 for regulatory T cells (Tregs) [6,7] which prevent severe autoimmune diseases throughout the lifespan of mice (and humans) by suppressing auto-reactive T cells that escape thymic negative selection [8]. IL-2 has consequently become of therapeutic interest for achieving auto- or transplantation tolerance [9,10].

The compilation and tissue distribution of the IL-2 receptor probably accounts for the distinct effects of IL-2 to drive both effector and suppressor functions. IL-2 preferentially binds to its high affinity receptor which is composed of the α- (CD25), β- (CD122) and common γ-chains (CD132) and with a lower affinity to the dimeric IL-2 receptor consisting of CD122 and CD132. The high affinity receptor is constitutively expressed on Tregs and rapidly upregulated on conventional T cells and NK cells upon activation, while resting NK cells and memory CD8 T cells constitutively carry the low affinity receptor [11]. This particular expression pattern of the IL-2 receptor can be exploited to preferentially target selected arms of the immune system by using different doses of IL-2. High dose bolus IL-2 is approved by the FDA to treat renal carcinoma and melanoma, while low dose IL-2 therapies are currently tested in patients suffering from chronic graft-versus host disease (GVHD) or diabetes [9,10,12]. High doses of IL-2, however, carry the risk of vascular leak syndrome [13] and low dose IL-2 therapy requires daily administration to obtain a therapeutic benefit.

Recently it was shown that the effect of IL-2 can be skewed either towards CD8/NK cells or towards Tregs by complexing it with distinct clones of mAb against IL-2 (IL-2 complexes) [14–16]. Due to their extended serum half-life, IL-2 complexes exhibit a high biological activity and thereby circumvent the major disadvantages of recombinant IL-2 therapy [17]. The α-IL-2 clone JES6-1A12 binds to a specific site on IL-2 that is relevant for its interaction with the low affinity β chain (CD122) of the IL-2 receptor, which is mainly expressed on resting NK and CD8 memory T cells, but preserves its binding to the high affinity α-chain (CD25) which is expressed at the highest levels on Tregs. Thereby the effect of IL-2 when bound to the complexes is preferentially directed towards CD25^high^ regulatory cells rather than CD122^+^ NK/CD8 memory cells [16,18]. IL-2/JES6-1A12 complexes have been shown to alleviate several immune disorders in rodents owing to the extensive expansion and activation of Tregs *in vivo*. IL-2 complexes ameliorated collagen-induced arthritis, delayed the onset of experimental autoimmune encephalomyelitis, alleviated allergic airway disease and induced long term survival of allogeneic pancreatic islet cells [15,19,20]. Furthermore, IL-2 complexes enhanced the stable engraftment of MHC-matched allogeneic BM in sublethally irradiated mice [21], but have not been explored in MHC-mismatched BMT models so far and neither in non-cytotoxic/irradiation-free BMT settings.

Recently our group demonstrated that the adoptive transfer of recipient Tregs obviates the need for cytoreductive conditioning (i.e. irradiation or cytotoxic drugs) in a fully allogeneic BMT model when given together with rapamycin and costimulation blockade (α-CD40L, CTLA4-Ig). This regimen induces durable mixed chimerism and tolerance to skin and heart allografts [22,23]. However, in terms of clinical translation it would be preferable if individualized cell therapies can be replaced by drugs that selectively expand and activate Tregs *in vivo*. Therefore, we investigated the ability of IL-2 complexes to promote the engraftment of fully mismatched BM under costimulation blockade.

## Material and Methods

### Ethics statement

All animals were treated according to European Union guidelines of animal care. All animal experiments were approved by the internal review board of the Medical University of Vienna and by the Austrian Ministry of Science and Research (permission number GZ 66.009/0230-II/3b/2011).

### Mice

Female C57BL/6 (H2^b^) and female BALB/c (H2^d^) mice were obtained from Charles River Laboratories (Sulzfeld, Germany). C57BL/6-FoxP3tm1Flv/J mice (Foxp3-mRFP reporter mice) [24] expressing a monomeric red fluorescent protein (mRFP) under control of Foxp3 promotor and B6.SJL-Ptprca Pepcb/BoyJ (CD45.1) mice were purchased from Jackson Laboratories (Maine, USA) and bred in our own facility. All mice were housed under specific pathogen free conditions and females were used between 6 and 10 weeks of age with a weight between 15-20g. Up to 5 animals were kept in individually ventilated polysulfone cages (Tecniplast, Italy) at a monitored temperature of 20–24°C, with humidity between 50–70%, a constant 12 hour light and dark cycle and at least 70 air changes per hour. The cages were bedded with decorticated aspen wood and enriched with nesting material (Abedd, Vienna, Austria). Animals were provided with sterilized water and rodent chow (Sniff, Soest, Germany) ad libitum. All surgeries were performed under general anesthesia employing a mixture of Ketamine (100mg/kg) and Xylazin (5mg/kg) intraperitoneally (i.p.). The concept of 3Rs (replacement, refinement and reduction) had a fundamental impact on study design of the approved ethical protocol. All efforts were made to minimize distress and group size. The number of mice in each specific group is provided in the figure legend.

### Preparation of IL-2 complexes

IL-2 complexes were prepared by incubating either 5μg recombinant mouse IL-2 (eBioscience, San Diego, CA) with 25μg purified α-mouse IL-2 (clone JES6-1A12) (BioXcell,West Lebanon, NH) or 1μg recombinant mouse IL-2 with 5μg purified α-mouse IL-2 for 30 min at 37°C. IL-2 complexes were administered i.p. in a final volume of 200μl. Exact dosing of IL-2 complexes is stated in the text and in the figure legends.

### In vitro activated Tregs

CD4^+^CD25^+^ cells were purified by magnetic bead separation using negative selection for CD4^+^ and subsequent positive selection for CD25^+^ by incubating CD4^+^ enriched cells with PE-conjugated α-CD25 (PC61) followed by α-PE microbeads (CD4^+^CD25^+^ Regulatory T-cell Isolation Kit; Miltenyi Biotec, Bergisch Gladbach, Germany). CD4^+^ CD25^+^ separated Tregs were cultured for five days in 12-well plates in RPMI 1640 media (Biochrome, Berlin, Germany) supplemented with 200U/ml IL-2 (Sigma), 10% FCS (Linaris, Dossenheim, Germany), PenStrep (100U Penicillin, 100μg Streptomycin/ml; Sigma), 10mM Hepes (MP Biomedicals), 1mM Sodium Pyruvat (Sigma), 1x non-essential amino acids (Sigma) and 10μM β-Mercaptoethanol (Sigma). The plates were pre-coated with 10μg/ml α-CD3 (145-2C11) (BioXcell) and 1μg/ml α-CD28 (37.51) (BioLegend) in PBS overnight at 4°C.

### IL-2 complex expanded Tregs

Naive C57BL/6 mice received IL-2 complexes (5μg IL-2 / 25μg α-IL.2) on three consecutive days. Two days after the last administration CD4^+^ CD25^+^ Tregs were isolated from spleen and lymph nodes by magnetic bead separation for use *in vitro* and *in vivo*.

### Bone marrow transplantation protocol

Groups of age-matched C57BL/6 recipients received costimulation blockade consisting of α-CD40L (CD154) mAb (MR1, 1 mg, d0) (BioXcell) with or without CTLA4-Ig (0.5 mg, d2) (Bristol Myers Squibb Pharmaceuticals, Princeton, NJ) and 20×10^6^ unseparated BM cells recovered from naïve BALB/c donors (d0). BM cells were collected in M199 medium (Sigma-Aldrich, St. Louis, MO) supplemented with 4μg/ml Gentamicin Sulfate (MP Biomedicals, Santa Ana, USA) and 10mM Hepes Buffer (MP Biomedicals) (BM medium). Selected groups were administered with a short course of rapamycin (0.1 mg, d-1, d0 and d2) (LC Laboratories, Woburn MA) and either received *in vitro* activated or IL-2 complex expanded Treg populations (3×10^6^) at the time of BMT or IL-2 complexes (d-4, -3 and -2; 5μg IL-2 / 25μg α-IL-2). Indicated groups of recipients were irradiated with 1Gy total body irradiation (TBI) prior to BMT (d-1) using a Xylon X-Ray unit [25]. Irradiated mice were additionally treated with or without IL-2 complexes (d3, d5; 1μg IL-2 / 5μg α-IL-2). All reagents were administered i.p. in a phosphate buffered solution (PBS) and BM cells were injected intravenously (i.v.) in BM medium.

### In vivo treatment of naïve mice

IL-2 complexes were administered i.p. to naïve C57BL/6 mice at days 0 (first day of administration), 1 and 2 in a final volume of 200μl [15]. Rapamycin (0.1mg) (LC Laboratories) and a mutated IL-15-Fc fusion protein competitively inhibiting IL15-triggered signals [26] (4μg) (Chimerigen, San Diego, CA) were given together with IL-2 complexes i.p. on d0, 1 and 2. α-IL-6 (MP520F3, 1mg) (BioXcell) was injected i.p. on d-1, 1 and 3. A single dose of the mAb against both MHC class II molecules I-A/I-E (M5/114, 1mg) (BioXcell) was administered i.p. on the first day (d0) of IL-2 complex treatment. All reagents were administered i.p in PBS.

### Mixed lymphocyte reaction

4×10^5^ splenocytes from naive C57BL/6 mice or mice treated with IL-2 complexes were co-cultured with 4×10^5^ irradiated BALB/c (allogeneic) or C57BL/6 (syngeneic) BM cells for 4 days. The proliferation was measured every day by staining Ki67 within NK cell and CD8 T cell population. The cells were cultured in RPMI 1640 media (Biochrome) supplemented with 10% FCS (Linaris), PenStrep (100U Penicillin, 100μg Streptomycin/ml; Sigma), 10mM Hepes (MP Biomedicals), 1mM Sodium Pyruvat (Sigma), 1x non-essential amino acids (Sigma) and 10μM β-Mercaptoethanol (Sigma).

### In vitro suppression assay

4×10^5^ responding splenocytes from congenic CD45.1 mice were stimulated *in vitro* with 10μg/ml α-CD3 (145-2C11) (BioXcell) for four days in in RPMI 1640 media (Biochrome, Berlin, Germany) supplemented with 10% FCS (Linaris, Dossenheim, Germany), PenStrep (100U Penicillin, 100μg Streptomycin/ml; Sigma), 10mM Hepes (MP Biomedicals), 1mM Sodium Pyruvat (Sigma), 1x non-essential amino acids (Sigma) and 10μM β-Mercaptoethanol (Sigma). 4×10^5^ *in vitro* activated or IL-2 complex (5μg IL-2 / 25μg α-IL-2) expanded Tregs from CD45.2 wildtype mice were added to selected wells. The proliferation of responding (CD45.1) CD4 and CD8 T cells was measured based on their expression of Ki67.

### Flow cytometry analysis

PerCP/Cy5.5 α-mouse CD3 antibody (17A2), APC/Cy7 α-mouse CD4 antibody (RM4-5), PE/Cy7 α-mouse CD8a antibody (53–6.7), FITC α-mouse/human Helios antibody (22F6), biotin α-mouse/human CD44 antibody (IM7), PE/Cy7 α-mouse CD25 antibody (PC61), biotin α-mouse H-2D^d^ antibody (34-2-12), FITC α-mouse/human CD11b antibody (M1/70), PE α-mouse CD19 antibody (6D5), FITC α-mouse NK-1.1 antibody (PK136) and PE α-mouse CD62L antibody were purchased from BioLegend (San Diego, CA). α-mouse Neuropilin-1 PE (761705) was acquired from R&D Systems (Minneapolis, MN). α-mouse/rat Foxp3 APC (FJK-16s) and α-mouse/rat Ki-67 PE-Cy7 (SolA15) were obtained from eBioscience. For intracellular staining the cells were permeabilized with the Foxp3/Transcription Factor Staining Buffer Set from eBioscience according to the manufacturer’s specification. Flow cytometric analysis was performed with a BD FACSCanto II or Beckman Coulter FC500 flow cytometer and data were analyzed by FlowJo (10.0.8) software.

### Epifluorescence microscopy

CD4^+^CD25^+^ cells were purified from Foxp3-mRFP reporter mice by magnetic bead separation and spun down onto a glass slide. The cell fluorescence was analyzed by a Zeiss LSM 510 Epifluorescence Microscope (magnification 63x) using a mercury-vapor lamp as light source.

### Statistical analysis

Ordinal variables were compared with a Fisher-exact test. A two-sided Student's t-test was used to compare percentage of donor cells within the myeloid lineage, mean fluorescent intensities (MFI) and absolute cell numbers. A p-value below 0.05 was considered statistically significant (* p < 0.5, ** p < 0.01, *** p < 0.001, **** p < 0.0001, n.s. p>0.5). Error Bars represent standard errors of the mean (SEM). Mean values were used to calculate fold changes. Data were statistically analyzed with GraphPad Prism 5.0.

## Results

### IL-2 complexes inhibit bone marrow engraftment

**Fig 1:**
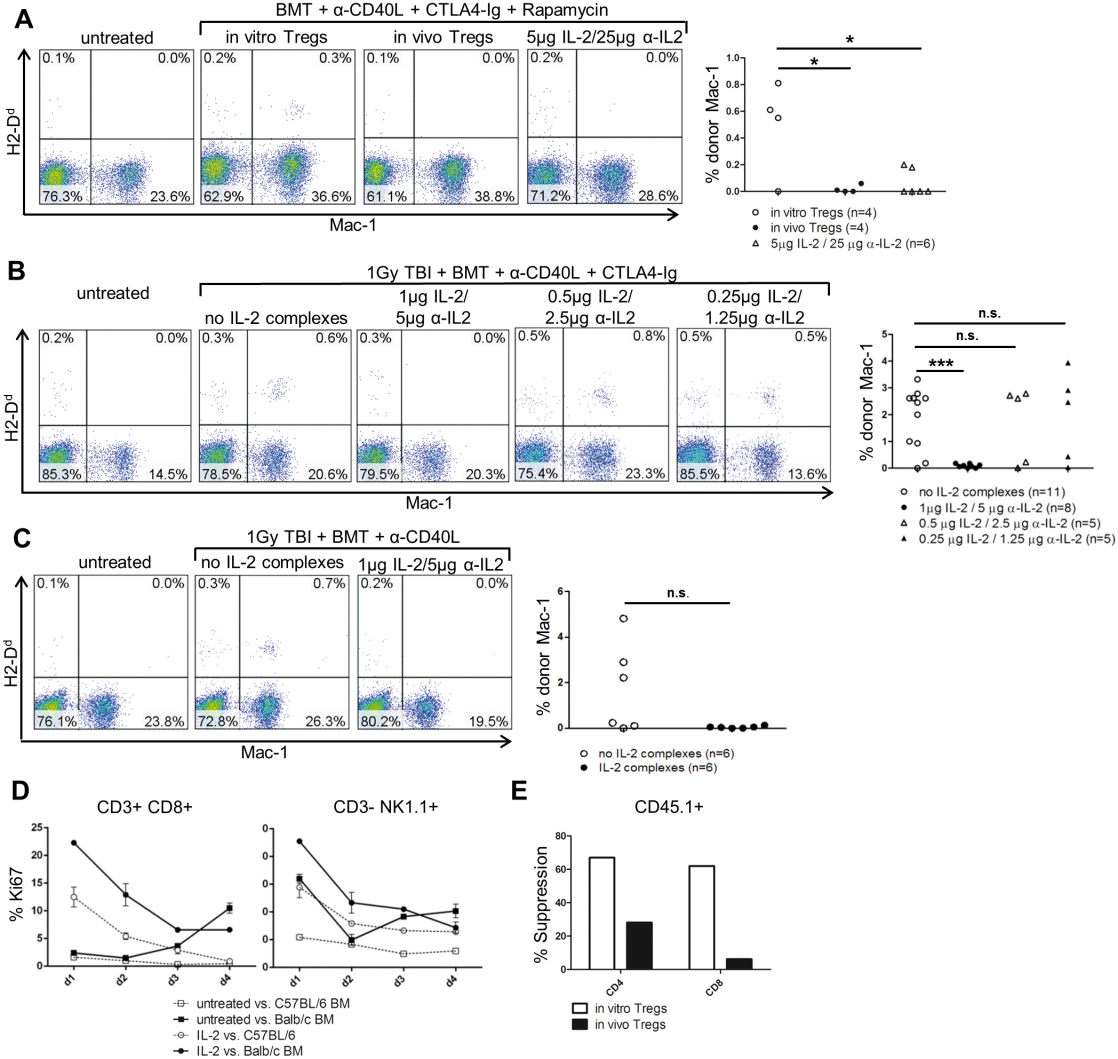
IL-2 complexes promote BM rejection. The ability of IL-2 complexes to replace Treg therapy was tested in different BMT models. Donor chimerism was analyzed in blood 14 days after transplantation by staining the BALB/c specific marker H2-D^d^ on myeloid (Mac-1) cells. **(A)** IL-2 complexes were less effective than Treg therapy in promoting BM engraftment. Naïve C57BL/6 mice were grafted with 20×10^6^ unseparated BALB/c BM cells (d0) under the cover of costimulation blockade (α-CD154, CTLA4Ig) and a short course of rapamycin. The recipients were additionally treated with either *in vitro* activated Tregs (3×10^6^) (n = 4), IL-2 complex expanded Tregs (3×10^6^) (n = 4) or IL-2 complexes (5μg IL-2 / 25μg α-IL-2; d-4, d-3, d-2) (n = 6). Two color flow cytometry plots are shown from representative BMT recipients (left). Each dot of the scatter diagram represents one mouse from one experiment (right) [*in vitro* Tregs vs. *in vivo* Tregs: p = 0.0341; *in vitro* Tregs vs. IL-2 complexes: p = 0.0187]. **(B)** IL-2 complexes enhanced BM rejection. Naïve C57BL/6 mice received a total body irradiation of 1Gy, costimulation blockade (α-CD154, CTLA4-Ig), as well as 20×10^6^ unseparated BALB/c BM cells (d0) with varying doses (1μg IL-2 / 5μg α-IL-2, n = 8; 0.5 μg IL-2 / 2.5μg α-IL-2, n = 5, 0.25 μg IL-2 / 1.25μg α-IL-2, n = 5) of IL-2 complexes; (d3, d5) or without IL-2 complexes (n = 11). Two-color flow cytometry plots are shown from representative BMT recipients (left). Each dot in the scatter diagram depicts one mouse from two individual experiments (right) [no IL-2 complexes vs. 1μg IL-2 / 5μg α-IL-2 p = 0.0004; no IL-2 complexes vs. 0.5μg IL-2 / 2.5μg α-IL-2: p = 0.7769, no IL-2 complexes vs. 0.25μg IL-2 / 1.25μg α-IL-2: p = 0.8966]. **(C)** Omission of CTLA4-Ig did not reverse the detrimental effect of IL-2 complexes. Naïve C57BL/6 mice were irradiated with 1Gy TBI before receiving costimulation blockade (α-CD154) and 20×10^6^ unseparated BALB/c BM cells (d0) with (1μg IL-2 / 5μg α-IL-2; d3, d5) (n = 6) or without IL-2 complexes (n = 6). Two-color flow cytometry plots are shown from representative BMT recipients (left). Each dot in the scatter diagram shows one mouse from one experiment (right) [p = 0.0627]. **(D)** IL-2 complexes increased the reactivity of CD8 T cells and NK cells toward donor antigens. Splenocytes from untreated mice or mice treated with IL-2 complexes were stimulated *in vitro* with irradiated BALB/c (allogeneic) or C57BL/6 (syngeneic) BM cells. The proliferation of CD8 T and NK cells was assessed by measuring the proliferation marker Ki67. Each symbol represents 2 mice from one experiment. **(F)** 4×10^5^ responder splenocytes from congenic CD45.1 mice were stimulated *in vitro* with α-CD3 and co-cultured with equal number of either *in vitro* activated or IL-2 complex expanded Tregs.

The adoptive transfer of polyclonal recipient Tregs (in vitro Tregs) is uniquely potent in promoting the engraftment of allogeneic BM in recipients conditioned only with rapamycin and costimulation blockade (without irradiation) [22,27]. To test whether IL-2 complexes can substitute Treg cell therapy in this setting, BMT recipients (C57BL/6) received fully MHC-mismatched BALB/c BM (20×10^6^ cells per mouse), costimulation blockade (α-CD154 mAb, CTLA4-Ig) and rapamycin together with either Treg transfer or IL2 complex (5μg IL2 + 25μg α-IL-2) treatment (4, 3 and 2 days before BMT). Unexpectedly, none of the mice receiving IL-2 complexes developed chimerism, whereas three of four mice treated with Tregs developed chimerism [0/6 vs. 3/4; p = 0.0333] (Fig 1A).

To avoid the potential stimulation of donor-reactive CD8 T and NK cells prior to transplantation, we next administered IL-2 complexes 3 and 5 days after BMT and also reduced the administered dose to a fifth (1μg IL-2 + 5 μg α-IL-2). This time, we conditioned the recipients with a regimen of non-myeloablative irradiation (1Gy TBI) and costimulation blockade that is effective without Treg therapy to distinguish between the two possibilities that either the suppressive capacity of the IL-2 complex was merely insufficient for establishing engraftment (in non-irradiated recipients) or that indeed IL-2 complexes actively promoted rejection of donor BM. Mice treated with IL-2 complexes again did not develop chimerism, while in contrast mice from the control group exhibited high levels of chimerism [0/8 with vs. 9/11 without IL-2 complexes, p = 0.0007] Further reduction of the administered dose (by 50% and 75%, respectively) also did not improve BM engraftment (Fig 1B).

To exclude the possibility that CTLA4-Ig impaired Tregs expansion or function under IL-2 complexes [28], we excluded the fusion protein from this non-myeloablative protocol (conditioning only with anti-CD40L and 1 Gy TBI). Again, none of the IL-2 complex treated mice developed chimerism [0/6 with vs. 3/6 without IL-2 complexes; p = 0.627] (Fig 1C).

To directly test whether IL-2 complexes enhance alloreactivity, we stimulated splenocytes from naïve or IL-2 complex treated mice *in vitro* with irradiated donor BM cells and analyzed proliferation based on the expression of Ki67 [29]. CD8 T and NK cells exhibited an increased rate of proliferation against allogeneic (BALB/c) BM when stimulated with IL-2 complexes [d1: mean % Ki67 (untreated vs. IL-2 complexes) of CD8^+^: 2.7% vs. 23.6% and NK1.1^+^: 32.0% vs. 45.5%] that was distinctly higher than against syngeneic (C57BL/6) implying that IL-2 complexes indeed reinforced alloreactivity (Fig 1D).

In addition, we isolated *in vivo* expanded Tregs (in vivo Tregs) from IL-2 complex (5μg IL-2 + 25μg α-IL-2) treated mice to assess their suppressive function *in vitro* and *in vivo*. IL-2 complex expanded Tregs were functional but less effective than *in vitro* activated Tregs in suppressing the proliferation of polyclonal activated T cells *in vitro* (Fig 1E). Moreover, in contrast to *in vitro* Tregs, *in vivo* expanded Tregs failed to induce BM engraftment when combined with costimulation blockade and rapamycin [0/4 vs. 3/4; p = 0.1429] (Fig 1A).

In summary, these results demonstrate that IL-2 complexes promote the rejection of donor BM, likely through the activation of CD8 T and NK cells, and therefore cannot replace Treg therapy for promoting engraftment of allogeneic BM.

### IL-2 complexes expand Tregs through proliferation in the thymus and the periphery

**Fig 2:**
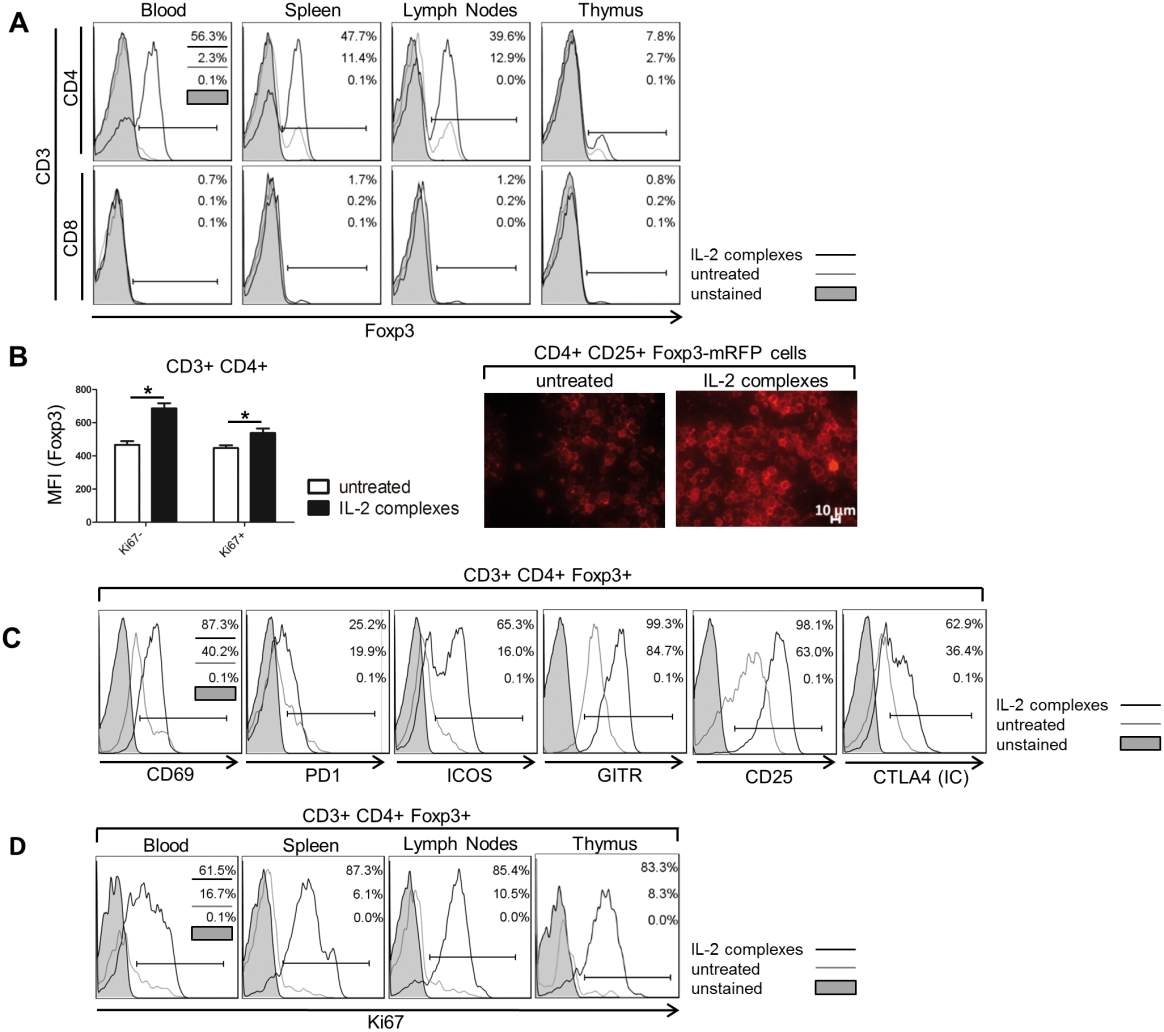
IL-2 complexes induce Treg proliferation in primary and secondary lymphoid tissues. The effectivity of IL-2 complexes to expand and activate Tregs *in vivo* was assessed. Female C57BL/6 mice were injected i.p. for 3 days (d0, d1, d2) with IL-2 complexes (5μg IL-2 / 25μg α-IL-2) or left untreated. Indicated tissues were analyzed 2 days after the last administration (d4) by flow cytometry. **(A)** IL-2 complexes increased the proportion of Foxp3^+^ Tregs within CD3^+^ CD4^+^ (upper panels) and CD3^+^ CD8^+^ T cells (lower panels) in blood, spleen, lymph nodes and thymus. Histograms display representative mice (n = 6). **(B)** Application of IL-2 complexes (n = 4) increased the intracellular expression of Foxp3 in comparison with untreated mice (n = 4) as determined by the MFI of Foxp3-APC [Ki67^+^: p = 0.0012; Ki67^−^: p = 0.0304]. We compared Foxp3 expression between Ki67^+^ proliferating and Ki67^−^ resting CD4 T cells to ensure that the measured effect was truly intrinsic and did not result from the increased cell mass of the dividing cells. Data are pooled from two independent experiments (left). The increase of Foxp3 was also evident by epifluorescence microscopy of CD4^+^ CD25^+^ separated cells purified from Foxp3-mRFP reporter mice (magnification 63x). Representative pictures are shown (right). **(C)** *In vivo* expanded Tregs exhibited an activated phenotype. IL-2 complexes increased the surface expression of CD69, PD1, ICOS, GITR and CD25 on CD4^+^ Tregs, as well as their intracellular expression of CTLA4. Representative mice are shown (n = 4). **(D)** CD4^+^ Tregs primarily expand through proliferation in the blood, spleen, lymph nodes and thymus upon IL-2 complex treatment as determined by their intracellular expression of the proliferation marker Ki67. Representative mice were chosen for histograms (n = 4).

To determine why the activation of CD8 T and NK cells prevailed over the suppression by Tregs we analyzed the origin and appearance of the expanding Tregs in naïve mice after treatment with IL-2 complexes (5μg IL2 + 25μg α-IL-2; d0, d1, d2). As previously reported [15], two days (d4) after the last administration, the percentage of CD3^+^ CD4^+^ Foxp3^+^ Tregs in blood, spleen, lymph nodes and thymus was increased several-fold with up to 55% of all CD4 T cells expressing Foxp3 [% Foxp3 within CD3^+^ CD4^+^ (untreated vs. IL-2 complexes) in spleen: 10.70±1.09 vs. 48.55±2.13, p < 0.0001 and in lymph nodes: 11.80±1.07 vs. 41.18±2.01, p<0.0001] (Fig 2A **upper panels**). Furthermore, treatment with IL-2 complexes significantly increased the intracellular level of Foxp3 expression within the CD4 T cell population, as assessed by flow cytometry and confocal microscopy of CD4^+^ CD25^+^ separated cells expressing mRFP under the control of Foxp3 promotor. The MFIs of Foxp3 were compared between resting (Ki67^−^) and proliferating (Ki67^+^) Tregs to avoid bias from the increased cell mass of the dividing cells (Fig 2B). The expanding Tregs exhibited an activated phenotype as determined by the up-regulation of distinct activation markers (i.e. CD69, CD25, GITR) (Fig 2C). Additionally, IL-2 complexes also caused a significant, although lesser, expansion of CD3^+^ CD8^+^ Foxp3^+^ T cells [% Foxp3 within CD3^+^ CD8^+^ (untreated vs. IL-2 complexes) in spleen: 0.20±0.02 vs. 1.64±0.69, p = 0.0072 and in lymph nodes: 0.21±0.1 vs. 1.12±0.49, p = 0.0129] (Fig 2A **lower panels**). IL-2 complexes substantially increased Treg proliferation, as assessed by Ki67 expression, in blood, secondary lymphoid organs and the thymus (Fig 2D). In conclusion, IL-2 complexes induce a substantial expansion of Tregs *in vivo* by stimulating their proliferation in the thymus and the periphery.

**Fig 3:**
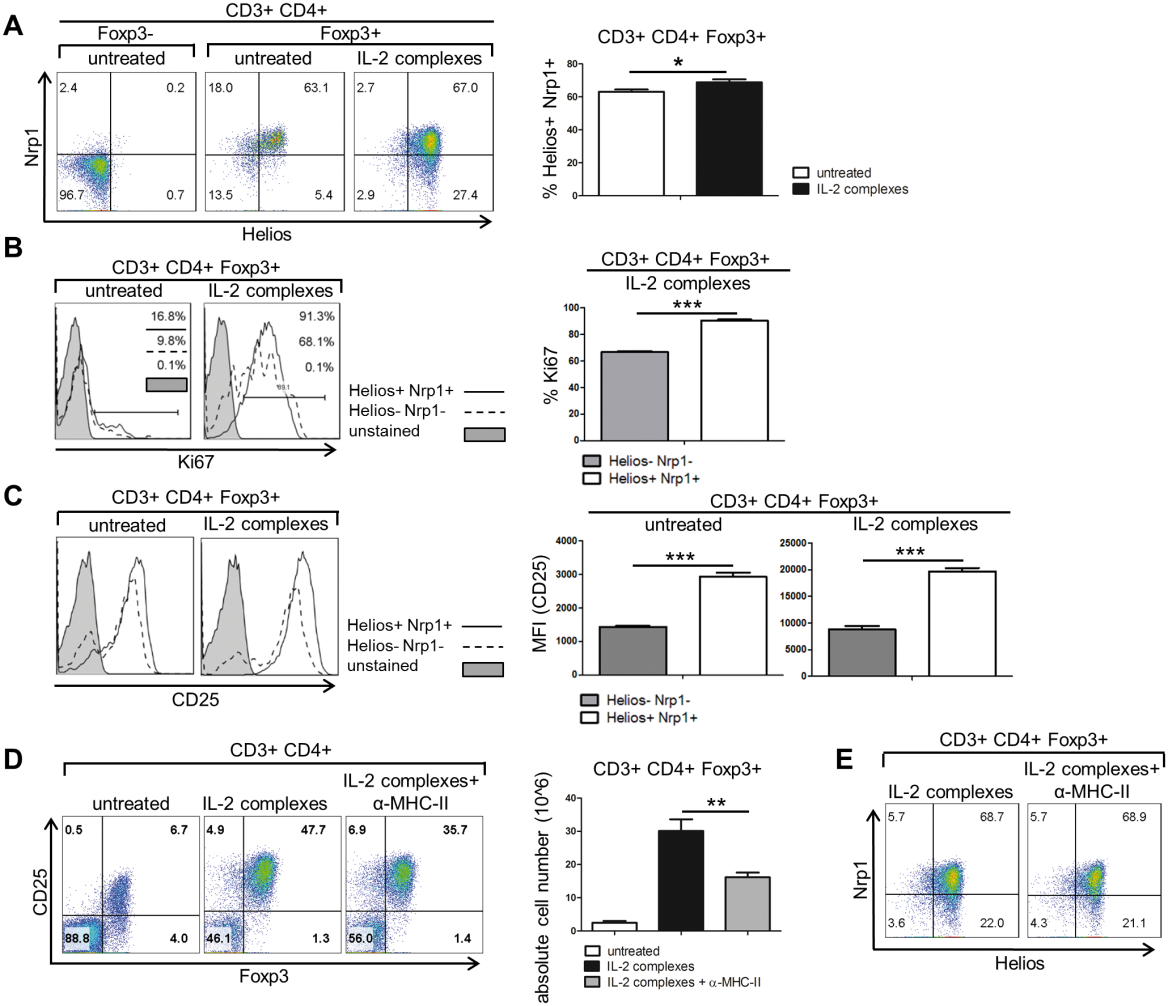
IL-2 complexes preferentially induce the proliferation of Helios^+^ Nrp1^+^ Tregs. To determine the effect of IL-2 complexes (5μg IL-2 / 25μg α-IL-2) on thymus derived Tregs, naïve C57BL/6 mice received three successive injections (d0, d1, d2) and Treg specific markers were measured in the spleen two days (d4) after the last injection. **(A)** IL-2 complexes (n = 6) slightly raised the amount of thymus derived Helios^+^ Nrp1^+^ Tregs compared to untreated mice (n = 6). Representative mice are displayed in two color dot plots (left). Data are pooled from three independent experiments (right) [p = 0.0287]. **(B)** Helios^+^ Nrp1^+^ Tregs exhibited a higher degree of proliferation than Helios^−^ Nrp1^−^ Tregs as measured by their expression of the proliferation marker Ki67 both before and after treatment with IL-2 complexes. Histograms show representative mice (left). The bars compare the expression of Ki67 between Helios^+^ Nrp1^+^ (n = 6) and Helios^−^ Nrp1^−^ (n = 6) Tregs upon IL-2 complex treatment (right) [p<0.0001]. Data are pooled from three independent experiments. **(C)** Helios^+^ Nrp1^+^ Tregs exhibit a higher surface expression of CD25. Representative mice were selected for histograms (left). The bars show the MFI of CD25-PE-Cy7 on Helios^+^ Nrp1^+^ and Helios- Nrp1- Tregs before (n = 3) and after (n = 3) IL-2 complex treatment (right) [untreated: p = 0.0003; IL-2 complexes: p = 0.0003]. The bars represent the mean of three mice from one experiment. **(D)** Blockade of MHC class II molecules decreased the amount and absolute cell number of CD4^+^ Tregs in the spleen compared to single giving of IL-2 complexes. Two color dot plots illustrate representative mice (left). Each bar shows the mean of 3 mice from one experiment (right) [p = 0.0031]. **(E)** Co-administration of α-MHC-II did not change the proportion of Helios^+^ Nrp1^+^ Tregs after IL-2 complex treatment. Representative mice are shown in the two color dot plots (n = 3).

We also analyzed the relative proportion of peripheral derived Tregs (pTregs) and thymus derived Tregs (tTregs) after treatment with IL-2 complexes. IL-2 complexes slightly but significantly increased the proportion of Helios^+^ Neuropilin-1^+^ (Nrp1) tTregs among Foxp3^+^CD4^+^ T cells [30,31] (Fig 3A). Helios^+^ Nrp1^+^ Tregs also exhibited a higher rate of proliferation in response to IL-2 complexes than their Helios^−^ Nrp1^−^ counterparts (Fig 3B). Helios^+^ Nrp1^+^ tTregs expressed higher levels of CD25 than double negative Tregs both before and after treatment with IL-2 complexes (Fig 3C), suggesting that the differential expression of CD25 accounts for the difference in the proliferative response between the two Treg subsets.

To address whether the increased proliferation of tTregs requires the interaction of their T cell receptors (TCRs) with self-antigens presented by MHC-II, we blocked MHC-II with an anti-I-A/ I-E mAb. Blocking MHC class II reduced the expansion of Tregs by approx. 25% and their absolute number by almost 50% but did not completely abolish it (Fig 3D). Since tTregs exhibit a higher affinity to self-peptide MHC-class II complexes than pTregs [32], we assumed that they would be more affected by the deprivation of a TCR signal. However, MHC class II blockade in the course of IL-2 complex treatment had no impact on the proportion of Helios^+^ Nrp1^+^ Tregs (Fig 3E). Hence, the TCR signal amplifies the expansion of p- and tTregs upon IL-2 complex treatment, but to a similar extent. These data provide evidence that in the course of IL-2 complex treatment signaling via the TCR promotes overall Treg proliferation, while the surface expression of CD25, which is higher in tTregs, determines the extent of proliferation.

### IL-2 complexes primarily target Tregs but also expand other lymphocytes

**Fig 4:**
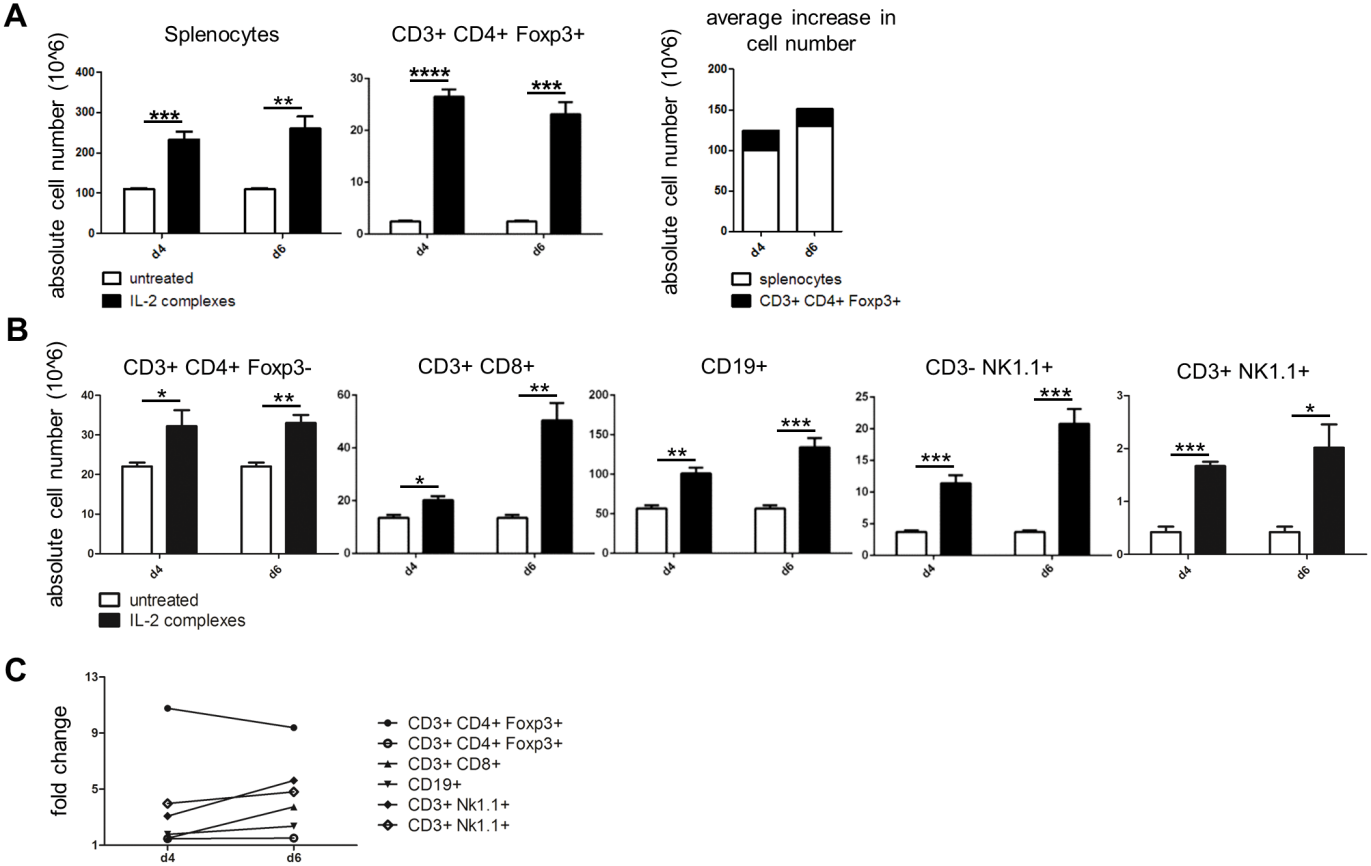
IL-2 complexes also expand lymphocyte populations other than Tregs. Distinct lymphocyte populations were analyzed in the spleen following IL-2 complex treatment to determine their specificity. Naïve C57BL/6 mice received IL-2 complexes (5μg IL-2 / 25μg α-IL-2) on three consecutive days (d0, d1, d2) and absolute cell numbers were analyzed 2 (d4) and 4 (d6) days after the last administration. **(A)** IL-2 complexes increased the absolute number of splenocytes [p = 0.0009 for d4; p = 0.0027 for d6] and splenic CD4^+^ Tregs [p <0.0001 for d4; p = 0.0001 for d6] 2 (d4) and 4 (d6) days after the last injection. The average increase in total splenocytes exceeds the mean increase of Tregs indicating that IL-2 complexes also expand other lymphocytes. Both groups consisted of 4 mice from 2 independent experiments at each time point. **(B)** IL-2 complexes also expand splenic CD3^+^ CD4^+^ Foxp3^−^ T cells [p = 0.0491 for d4; p = 0.0028 for d6], CD3^+^ CD8^+^ T cells [p = 0.0118 for d4; p = 0.0016 for d6], CD3^−^ NK1.1^+^ NK cells [p = 0.0009 for d4, p = 0.0004 for d6], CD3^+^ NK1.1^+^ NK cells [p < 0.0001 for d4, p = 0.0012 for d6] and CD19^+^ B cells [p = 0.0016 for d4, p = 0.008 for d6]. Both groups consisted of 4 mice respectively at each time point. Data are pooled from 2 independent experiments. **(C)** Fold change of distinct lymphocyte populations in the spleen at d4 and d6.

As the effect of IL-2 complexes was not exclusively restricted to Tregs we aimed to assess the expansion of other lymphocyte populations. In line with other reports, treatment with IL-2 complexes caused severe splenomegaly and lymphadenopathy in otherwise naïve mice [14], with the total number of splenocytes increasing by 120 million cells on average (i.e. two-fold). Tregs, which increased by more than 20 million cells (ten-fold), accounted for only a sixth of the overall cell increase (Fig 4A). IL-2 complexes also significantly increased the absolute numbers of conventional CD4^+^ Foxp3^−^ T cells, CD8 T cells, NK cells, NKT cells and B cells, which reached the peak of expansion on day 6 (up to five-fold expansion) (Fig 4B and 4C). In contrast, Tregs exhibited a ten-fold increase that peaked already on day 4 (Fig 4C). Thus, although IL-2 complexes preferentially target Tregs they also expand other lymphocyte subsets in naïve hosts, albeit to a lesser extent.

### IL-2 complexes mobilize B cells from the bone marrow

**Fig 5:**
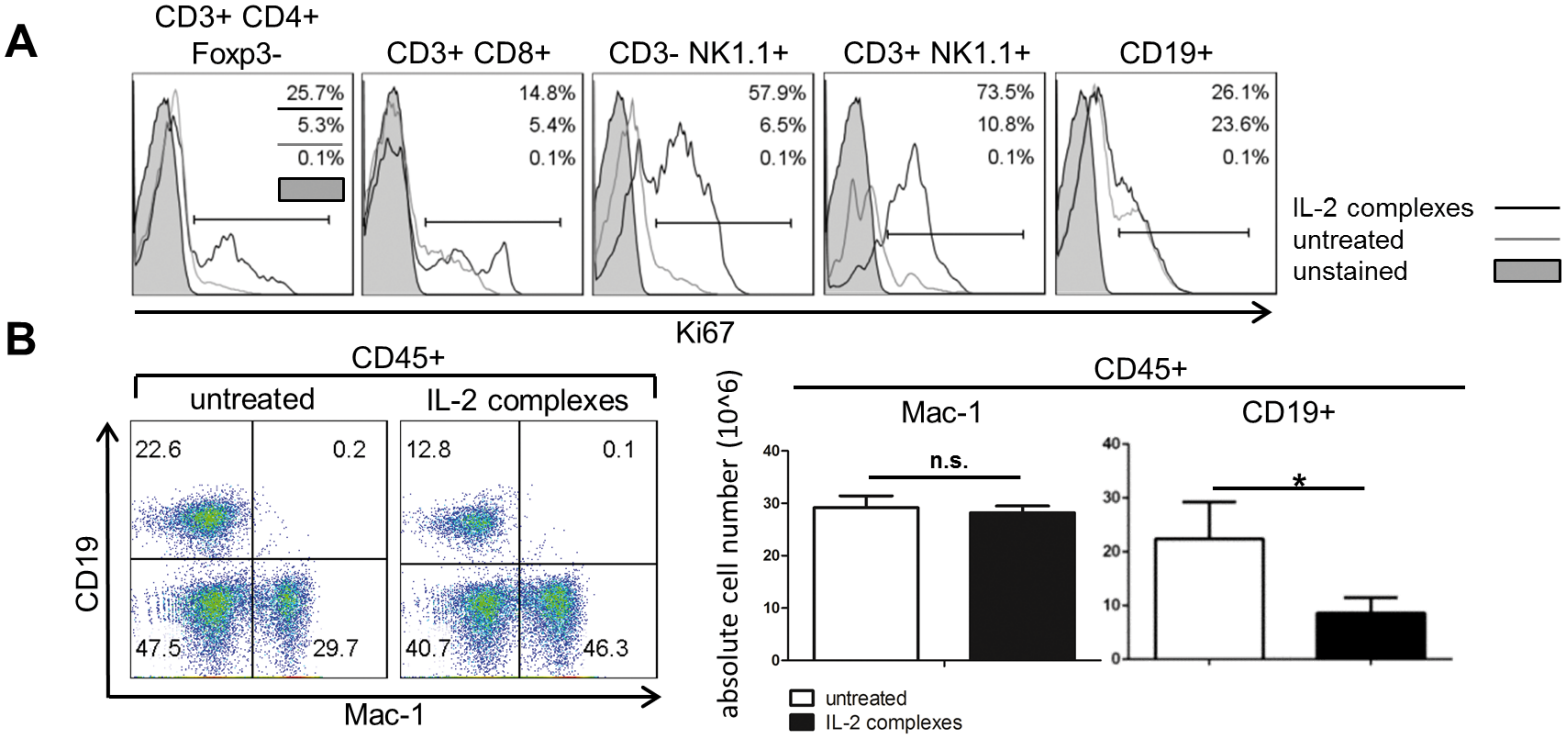
IL-2 complexes relocate B cells from the bone marrow to secondary lymphoid organs. The mechanisms of B cell expansion in secondary lymphoid organs were investigated. Naive C57BL/6 mice received IL-2 complexes (5μg IL-2 / 25μg α-IL-2) for three days (d0, d1, d2) and distinct cell populations were analyzed in indicated tissues 2 days (d4) after the last dose. **(A)** CD3^+^ CD4^+^ Foxp3^−^ T cells, CD3^+^ CD8^+^ T cells, CD3^−^ Nk1.1^+^ NK cells and CD3^+^ NK1.1^+^ cells but not CD19^+^ B cells proliferated upon IL-2 complex treatment in the spleen as assessed by their expression of Ki67. Histograms are shown from representative mice (n = 4). **(B)** IL-2 complexes (n = 4) reduced the proportion and absolute number of CD19^+^ B cells within CD45^+^ leukocytes in the BM as compared with untreated mice (n = 4). Two color dot plots illustrate representative mice (left). Data are pooled from 2 independent experiments (right) [p = 0.014].

IL-2 complexes likely induce the proliferation of CD8 T cells and NK cells directly through CD122 expressed on these subsets. B cells also strongly expanded even though only very few (~1%) B cells in the periphery express either the dimeric (CD132, CD122) or trimeric (CD132, CD122, CD25) IL-2 receptor (data not shown and [33]). Moreover, IL-2 complexes increased the number of proliferating conventional CD4 T cells, CD8 T cells and NK cells but had no significant effect on the proportion of proliferating B cells in the spleen (Fig 5A). As most CD25^+^ B cells are located in the BM (data not shown and [33]), we measured the proportion and absolute number of CD19^+^ B cells in the BM and found that both were significantly decreased upon treatment with IL-2 complexes [proportion of CD19^+^ B cells within CD45^+^ leukocyte population in the BM (naïve vs. IL-2 complexes): 25.8% ± 7.7 vs. 13.9% ±4.3, p = 0.03)] (Fig 5B). Taken together, these data suggest that IL-2 complexes stimulate B cells to relocate from the BM to secondary lymphoid organs such as the spleen.

### Rapamycin attenuates the expansion and activation of CD8 T cells

**Fig 6:**
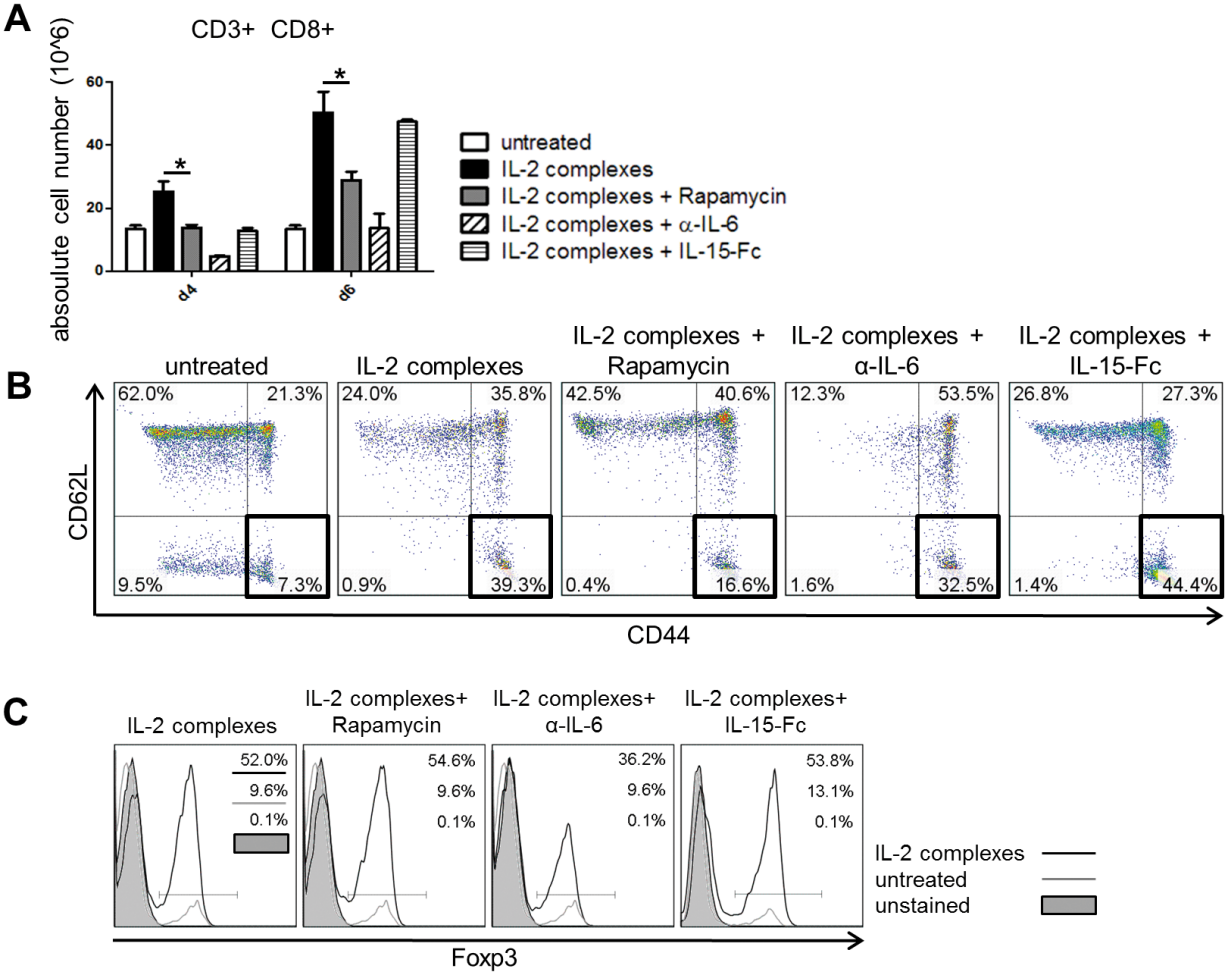
Rapamycin reduces the expansion and activation of CD8 effector cells. IL-2 complexes were combined with selected agents to alleviate the unintended expansion and activation of CD8 T cells. IL-2 complexes (5μg IL-2 / 25μg α-IL-2) were administered to female C57BL/6 mice three times in succession (d0, d1,0 d2) together with specified agents. The effect of combined therapy was determined on splenic CD8^+^ T cells and CD4^+^ Foxp3^+^ Tregs 2 (d4) and 4 (d6) days after the last injection. **(A)** Co-administration of rapamycin (n = 4) reduced the absolute cell number of splenic CD8^+^ T cells at both measured time points compared to mice treated only with IL-2 complexes (n = 4) [p = 0.0142 for d4; p = 0.0244 for d6]. Data are pooled from two independent experiments. Groups receiving α -IL-6 and IL-15-Fc consisted of 2 mice from one experiment at each time point. **(B)** Rapamycin (n = 4) but not α-IL-6 (n = 2) or IL-15-Fc (n = 2) considerably decreased the amount of effector memory CD8^+^ T cells (d6) defined as CD44^hi^ CD62^lo^ compared to IL-2 complex treatment alone (n = 4). Two color dot plots depict representative mice. **(C)** Addition of α-IL-6 (n = 2) decreased the fraction of Foxp3^+^ cells within splenic CD4 T cell population while rapamycin (n = 4) and IL-15-Fc (n = 2) had no profound effect (d6). Histograms illustrate representative mice of each group.

Next, we determined whether the selectivity of IL-2 complexes could be improved through the co-administration of additional therapeutics reducing the expansion of CD8 T cells while leaving Treg expansion unperturbed. For this purpose, we selected the mTOR inhibitor rapamycin [34], an anti-IL-6 mAb (α-IL6) and an antagonistic mutated IL-15-fusion protein (IL-15-Fc) [26]. Rapamycin promotes the expansion of Tregs while inhibiting the differentiation and proliferation of effector T cells [35]. Furthermore, rapamycin—which is part of the BMT protocol using Treg therapy—has recently been shown to synergize with IL-2 complexes in steering the immune response towards regulation [15,36]. IL-6 has been shown to play a decisive role in determining whether naïve T cells differentiate into Tregs or pro-inflammatory Th17 cells [37]. Therefore we assumed that blocking IL-6 [38]would direct the effect of IL-2 complexes more towards regulation than inflammation. As IL-15 competes with IL-2 for the dimeric low-affinity IL-2 receptor (CD122, CD132), but not the high affinity receptor (CD25, CD122, CD132), we anticipated that administration of a mutated, antagonistic IL-15-Fc would reduce the effect of IL-2 complexes on CD122^+^ cells. In line with this, IL-15-Fc promotes tolerance induction when combined with an IL-2 fusion protein [39,40]. Co-administration of rapamycin or α-IL-6 with IL-2 complexes significantly reduced the expansion of CD8 T cells, while IL-15-Fc had no detectable effect 4 days after the last administration of IL-2 complexes (Fig 6A). IL-2 complexes led to an increased proportion of (central and effector) memory phenotype CD8 T cells (CD44^hi^ CD62L^hi^ or CD62L^lo^, respectively). Rapamycin (but not α-IL6 or IL-15-Fc) decreased the proportion of effector memory CD8 T cells (CD44^hi^ CD62L^lo^) [% CD44^hi^ CD62L^lo^ among CD8 T cells (IL-2 complexes vs. IL-2 complexes + rapamycin): 39.3% vs. 16.6%] (Fig 6B). Rapamycin and IL-15-Fc had no clear effect on the fraction of Tregs, whereas α-IL-6 mAb unexpectedly reduced Treg expansion by one third (Fig 6C). In summary, it appears that rapamycin reduces the expansion and activation of CD8 T cells, while simultaneously preserving the proliferation of Tregs. Thus, beyond rapamycin, which is already part of our irradiation-free BMT protocol, neither IL-15-Fc nor α-IL6 seems to provide a mechanistic rationale for combination with IL2 complexes to increase specificity.

## Discussion

Modulating the balance between regulatory and effector T cells is of high interest for the treatment of immunological disorders. While individualized regulatory cell therapy shows potent effects in pre-clinical studies, clinical translation faces substantial hurdles [41,42]. Treg-specific pharmacologic therapies with small molecules or biologics would be a highly attractive alternative.

In the present study, we explored whether treatment with IL-2 complexes could replace the transfer of Tregs which is essential for achieving BM engraftment in an irradiation-free setting. Our results provide evidence that IL-2 complexes enhance rather than alleviate alloreactivity towards BM actually triggering its rejection. This unintended effect was not abolished by delaying IL-2 complex administration until after BMT, which was beneficial in a sublethal MHC-matched BMT model [21]. Substantially lowering the IL-2 complex dose, which was effective in preventing diabetes in pre-diabetic NOD mice [43], still was unable to promote BM engraftment. As CTLA4-Ig abrogated the therapeutic effect of IL-2 complexes in a murine MHC class II mismatched skin graft model [44], we omitted the fusion protein from our protocol, but nevertheless did not detect a beneficial effect of IL-2 complexes.

We tested the IL-2 complexes in two BMT models. First we deployed an irradiation-free non-cytotoxic one in which BM engraftment is critically dependent on Treg therapy [22] to directly compare the efficacy of IL-2 complexes with that of adoptively transferred Tregs. Further, we used a non-myeloablative model where Treg therapy is not critically required but improves BM engraftment [45] in order to determine whether IL-2 complexes are just less effective than Treg therapy or actively promote BM rejection. In both models, IL-2 complexes failed to induce BM engraftment but instead triggered its rejection. Two factors were identified that are likely to contribute to this effect: the unintended activation of CD8 T and NK cells and the reduced suppressor function of IL-2 complex expanded Tregs. Collectively, these results argue that IL-2 complexes cannot be substituted for Treg cell therapy in a fully MHC-mismatched, non-cytotoxic BMT model.

IL-2 complexes consisting of the α-IL-2 clone JES6-1A12 were originally described to primarily expand Tregs and to only modestly increase the number of CD122 positive CD8 T and NK cells [15]. Another group investigated the effect of IL-2 complexes on the proportion of B and myeloid cells without detecting significant differences [46]. Nevertheless, given the absolute cell numbers of Treg expansion, the massive splenomegaly and lymphadenopathy observed in mice treated with IL-2 complexes [14] cannot be solely explained by the expansion of Tregs. Intriguingly, patients receiving IL-2 immunotherapy as cancer treatment also feature a splenic enlargement [47]. Therefore we analyzed in detail the absolute cell numbers of distinct lymphocyte subsets in secondary lymphoid organs. Conventional CD4 T cells, CD8 T cells, NK cells, NKT cells and even B cells expanded after treatment with IL-2 complexes. Conventional CD4 T cells, CD8 T cells, NK cells, NKT cells proliferated in peripheral tissues upon IL-2 complex treatment although predominantly expressing the low-affinity IL-2 receptor. As B cells did not proliferate in the periphery but were significantly reduced in the BM, we concluded that IL-2 complexes induce the migration of B cells from the BM to secondary lymphoid organs, which is consistent with the fact that CD25 expression constitutes a crucial step in pre-B cell development [48] and IL-2^-/-^ mice exhibit a disrupted B cell development and survival [49].

However, from all expanding cell populations we were most concerned about CD8 T cells as they embody crucial mediators of acute BM rejection [50], and because their proliferation in response to alloantigens was particularly sensitive to IL-2 complexes (Fig 1D) and IL-2 expanded Tregs seem less effective in suppressing CD8 than CD4 cells *in vitro* (Fig 1E). Therefore we intended to inhibit their expansion by co-administration of other reagents. In contrast to IL15-Fc, α-IL-6 and rapamycin reduced the number of expanding CD8 T cells at both measured time points. Rapamycin was, however, the only agent to preserve the proliferation of Tregs under IL-2 complexes. The synergistic effect of rapamycin and IL-2 complexes has already been shown to ameliorate EAE, to promote the expansion of suppressive CD8^+^ Foxp3^+^ cells after BMT and to stabilize *in vitro* induced Tregs in a GVHD model [15,36,51]. Rapamycin presumably steers the effect of IL-2 complexes towards Tregs by inhibiting the expression of CD25 on effector T cells [52]. However, this effect of rapamycin was evidently insufficient to reverse the deleterious effect of IL-2 complexes in this specific BMT setting. Rapamycin efficiently inhibits the proliferation of NK cells but only has a modest effect on their IFN-γ secretion or cytolytic activity [34]. Accordingly, we speculate that IL-2 complexes enhanced rapamycin-resistant NK cell mediated donor BM rejection [53].

The effect of IL-2 complexes was partially prevented through the blockade of MHC class II, decreasing pTregs and tTreg expansion to a similar degree, suggesting that TCR signals increase the turnover of Tregs without, however, affecting the ratio between p- and tTregs. In accordance with this observation, the administration of IL-2 complexes could partially restore the proliferation of adoptively transferred Tregs in a MHC class II deficient host [54], obviating the need of a TCR signal for Treg proliferation to a certain degree.

The non-cytotoxic BMT protocols yield very low levels of chimerism (~1% myeloid donor cells in the blood after 2 weeks) wherefore the rejection of only a small number of donor cells may be sufficient to lead to graft failure. In such a stringent model the unintended activation of effector cells might thus be more devastating than in sublethal models where higher levels of chimerism are achieved [21]. In such models the loss of some donor BM cells can more readily be withstood and does not immediately lead to engraftment failure. Under these circumstances the beneficial effects of IL-2 might then take hold. Besides, the absence of MHC disparities prevents direct allorecognition by T cells and circumvents NK cell mediated rejection which is likewise a MHC dependent process [55]. Under these circumstances the beneficial effects of IL-2 complexes are more likely to emerge.

IL-2 complexes also averted the rejection of fully MHC–incompatible pancreatic islets [15]. We assume that the unintended activation of CD8 T and NK cells through IL-2 complexes is more detrimental in our BMT model as BM cells infused intravenously (as opposed to islet cells transplanted under the kidney capsule) are exquisitely sensitive to rejection through CD8 T cells and NK cells in the spleen before they home to the BM.

In summary this study indicates that IL-2 complexes preferentially induce the proliferation of Tregs but their effect is not sufficiently specific to replace the adoptive transfer of recipient Tregs in a non-cytoreductive BMT model.
